# Effect of brief smoking cessation online training on knowledge, attitudes, behaviors, and organizational factors in European health professions students: A pre–post study

**DOI:** 10.18332/tid/217007

**Published:** 2026-07-20

**Authors:** Cristina Martínez, Miren Idoia Pardavila-Belio, Kenza Laroussy, Carmen Moreno, Montse Puig, Zaida Agüeda, Raúl Sancho, Teresa Lluch, Navidad Canga-Armayor, María Pueyo-Garrigues, Sandra Tricas-Sauras, María Lavilla-Gracia, Maria J. Duaso, Jordi Vilaplana, Juidth Roca, Merçe Margalef, Esteve Fernández, Ariadna Feliu, INSTrUCT Research Group

**Affiliations:** 1 WHO Collaborating Centre for Tobacco Control, Institut Català d’Oncologia – ICO, Barcelona, Spain; 2 Tobacco Control Research Group, Institut d’Investigació Biomèdica de Bellvitge – IDIBELL, Barcelona, Spain; 3 Faculty of Nursing, University of Barcelona, Barcelona, Spain; 4 Centro de Investigación Biomédica en Red de Enfermedades Respiratorias – CIBERES, Madrid, Spain; 5 Philip R. Lee Institute for Health Policy Studies, University of California San Francisco, San Francisco, United States; 6 Department of Community, Maternity and Pediatric Nursing, School of Nursing, University of Navarra, Pamplona, Spain; 7 IdiSNA, Navarra Institute for Health Research, Pamplona, Spain; 8 Social Approaches to Health Research Center (CRISS-CR5), School of Public Health, Brussels, Belgium; 9 Florence Nightingale Faculty of Nursing, Midwifery & Palliative Care, King’s College London, London, United Kingdom; 10 Computer Science Department, Universitat de Lleida, Lleida, Spain; 11 Department of Nursing and Physiotherapy, Faculty of Nursing and Physiotherapy, University of Lleida, Lleida, Spain; 12 Health Care Research Group (GRECS), Biomedical Research Institute of Lleida, Lleida, Spain; 13 School of Medicine and Health Sciences, University of Barcelona, Barcelona, Spain; 14 Catalan Health Department, Government of Catalonia, Barcelona, Spain; 15 Environment and Lifestyle Epidemiology Branch, International Agency for Research on Cancer (IARC), World Health Organization (WHO), Lyon, France

**Keywords:** health occupations, education, tobacco control, students, implementation science

## Abstract

**INTRODUCTION:**

Health professionals play a critical role in tobacco prevention and cessation, but limited training during education often leaves their knowledge, attitudes, behaviors, and organizational support insufficient. Our aim was to assess the effect of an online brief smoking cessation training program on knowledge, attitudes, behaviors, and organizational factors among health professions students in Spain and the United Kingdom.

**METHODS:**

A pre-post study was conducted among 756 undergraduate health professions students (652 from Spain and 104 from the UK) from four universities. Participants completed the validated Knowledge, Attitudes, Behaviors, and Organizational factors self-reported questionnaire for health professions Students (KABO_S) before and one month after the training. Generalized linear mixed models analyzed pre-post changes, adjusting for gender, age, and tobacco use.

**RESULTS:**

Significant increases were observed in the overall KABO_S score from 4.82 (SD=1.34) to 6.69 (SD=1.26) at one month (p<0.01). All dimensions increased in both countries (all p<0.01), with the greatest gains in individual knowledge/skills and attitudes/beliefs (median improvements ranging from 38.5% to 69.1%). Organizational resources showed the lowest gains (median improvement of 5.7% to 15.2%). Linear mixed models confirmed a significant increase in overall KABO_S scores (Spain: β=1.78; 95% CI: 1.67–1.90; UK: β=2.44; 95% CI: 2.13–2.74) and in each individual dimension (all p<0.001).

**CONCLUSIONS:**

Knowledge, attitudes, behaviors, and organizational factors improved significantly among health professions students in both countries. These findings suggest that this online training program may represent a promising addition to health professions curricula in Europe.

## INTRODUCTION

The prevalence of global tobacco use has declined significantly in recent decades^1^. However, in 2021, 21.7% of individuals aged ≥15 years in WHO Member States worldwide still reported using tobacco products^2^. The WHO European Region continues to have the highest adult smoking prevalence (28%) globally, including some of the highest prevalence rates among health professionals, reaching up to 46% in certain countries^3,4^.

Health professionals play a critical role in tobacco control, particularly through the delivery of smoking cessation interventions, one of the most cost-effective strategies to reduce tobacco use at the population level^5,6^. Fewer than 7% of smokers successfully quit smoking without assistance; however, receiving advice and support from a health professional more than doubles the likelihood of quitting successfully. This likelihood increases further when pharmacological treatments are incorporated into the quit plan^7-9^. Despite this, although about 70% of smokers have contact with a health professional annually, only 29.1% are offered cessation counselling or pharmacological support^7-8^. These figures highlight a major missed opportunity to deliver effective interventions at scale.

Given the lack of adequate training, the WHO Framework Convention on Tobacco Control (FCTC) strongly advocates for health professionals’ appropriate training in the effective management and treatment of tobacco dependence^5^. Such training should include/involve comprehensive educational programs aimed at developing the key competencies – knowledge, attitudes, behaviors, and skills – needed to deliver personalized cessation support^10,11^. However, studies consistently show that smoking cessation is often inadequately addressed in health professions curricula worldwide^12,13^. In nursing education specifically, smoking cessation remains under-represented, with limited curricular time and few opportunities for applied learning and clinical skill development^12^. As a result, both health professionals and students frequently report feeling unprepared and lacking confidence in delivering smoking cessation interventions, with inadequate training cited as a key barrier^14-16^. Addressing this gap, higher education represents a critical window not only for educating future professionals about the harms of tobacco use, but also for shaping positive attitudes and building the competencies required to implement effective cessation interventions^17^.

Several educational initiatives have emerged to enhance smoking cessation-related competencies among health professions students. One such initiative is the INSTrUCT project which developed a comprehensive, evidence-based online training program aimed at integrating smoking cessation content into higher education curricula across Europe. The INSTrUCT Online Brief Intervention for Smoking Cessation Training Program is designed to improve students’ knowledge, motivation, self-efficacy, and practical skills^18^. Unlike traditional programs, it addresses individual attitudes and behavioral competencies alongside knowledge offering a wide range of applied and interactive content^19^. The program has already been successfully implemented in four universities across Europe, with over 86% of students reporting they acquired the necessary competencies to deliver smoking cessation interventions. It is also designed for easy integration into health professions curricula, offering free, ready-to-use materials and comprehensive instructor guidance^18^.

Previous evaluations have demonstrated the effectiveness of this program in supporting health professions students to acquire smoking cessation competencies, particularly in terms of knowledge, attitudes, and skills^18^. However, further evidence is needed from a more detailed pre-post analysis using a validated multidimensional instrument (KABO_S: Knowledge, Attitudes, Behaviors and Organization questionnaire for Students) that also incorporates perceptions of organizational support and resources^17^. It uniquely examines both behavioral and contextual changes, as well as the program’s integration into diverse academic environments. Therefore, this study aims to assess the effect of an online brief smoking cessation training program on knowledge, attitudes, behaviors, and organizational factors among health professions students in Spain and the United Kingdom (UK).

## METHODS

### Design and setting

An interventional pre-post study was conducted within the INSTrUCT project, which initially involved seven universities across four European countries (Spain, the UK, Belgium, and Portugal). For the present analysis, the study was conducted among health professions students at four universities, three in Spain and one in the UK: the University of Barcelona, the University of Lleida, the University of Navarra, and King’s College London.

### Participants and recruitment

Participants were undergraduate students enrolled in health professions degrees as part of the INSTrUCT project. These degrees included nursing, medicine, and other related fields (pharmacy and psychology). In total, 1072 students from seven universities in four European countries (Spain, the UK, Belgium, and Portugal) were recruited between January 2020 and June 2022. Recruitment was conducted via classroom announcements, email invitations, and coordination by local lecturers at each university, who also served as points of contact throughout the study^18^.

**Figure 1:**
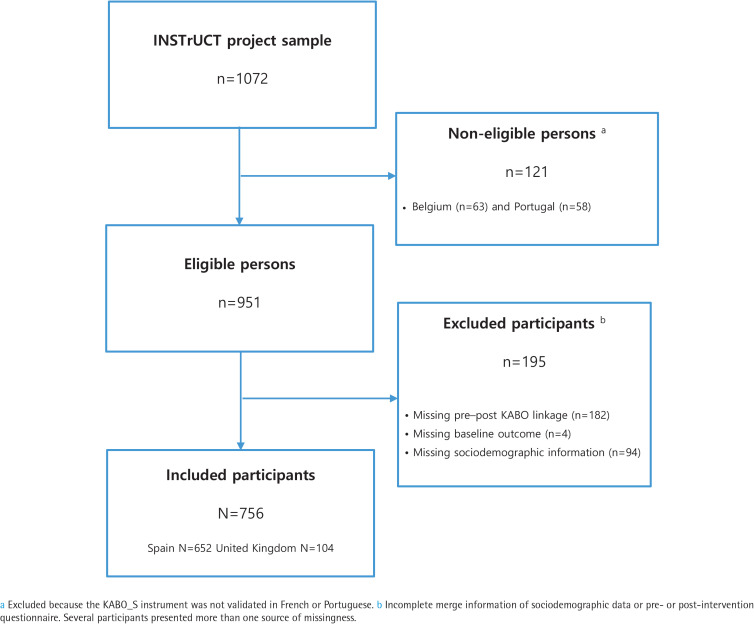
Participant recruitment flow diagram for a pre–post study of undergraduate health professions students in Spain (N=652) and the United Kingdom (N=104), January 2020–June 2022

For the present analysis (analytic sample), only the subset of students from Spain and the UK were included (N=756). This was because the KABO_S evaluation instrument had been validated only in Spanish and English, and therefore students from Belgium (n=63) and Portugal (n=58) were excluded due to the lack of validated versions in French and Portuguese (Figure 1).

Students were invited to participate voluntarily after receiving information about the study’s objectives, design, and methodology. Following eligibility assessment, 195 participants were excluded, primarily due to failure to meet data quality criteria for key merge variables required to link pre- and post-intervention KABO_S questionnaires (n=182), missing baseline outcome data (n=4), or missing sociodemographic information (n=94). Most excluded participants presented multiple sources of missingness. To assess potential attrition bias, sociodemographic characteristics were compared between included (n=756) and excluded (n=195) participants for whom information was available. Excluded participants were older on average than those included (mean age 23.7 years, SD=7.1 vs 21.6 years, SD=4.8), and nearly half did not report their sex (48.7%), indicating that exclusions were largely driven by structural missingness in sociodemographic data rather than by loss to follow-up, although some degree of attrition bias cannot be entirely ruled out (Figure 1). The final sample was 756 (Spain n=652; UK n=104).

### Intervention description

The INSTrUCT Online Brief Intervention for Smoking Cessation Training Program is a self-directed, competency-based educational resource designed to equip future health professionals with the skills and knowledge necessary to deliver brief smoking cessation interventions. The program consisted of five theoretical modules (approximately 5 hours), five educational videos (1–2 hours), and three virtual simulation cases (1–2 hours). Although the learning management system verified completion of all modules, videos, and simulation cases, it did not capture component-specific time-on-task data. Consequently, the precise number of hours devoted to training by individual students could not be ascertained. Nevertheless, based on pilot testing, students were expected to invest between 19 and 21 hours in independent study, in addition to approximately two hours allocated to the final assessment^18^.

The theoretical modules covered a range of topics, including the epidemiology of tobacco use, general concepts of tobacco and nicotine dependence, assessment of dependence and motivation to quit, and evidence-based cessation interventions – focusing on the brief intervention, as well as psychological and pharmacological treatment approaches. The educational videos showcased diverse clinical cases, aiming to contextualize the theoretical content and support observational learning. Finally, the three virtual simulations delivered through an interactive platform, where students engage with scripted patient interactions to apply the 5As model (Ask, Advise, Assess, Assist, Arrange), make clinical decisions, and receive automated feedback^18^.

### Instrument

The Knowledge, Attitudes, Behaviors and Organization questionnaire for Students (KABO_S) is a self-reported instrument designed to assess key factors influencing the implementation of smoking cessation interventions. It was originally developed for health professionals (KABO)^20^ and later adapted and validated for health professions students from Spain and the UK contexts (KABO_S)^17^.

The KABO_S consists of 17 items rated on an 11-point Likert-type scale that are grouped into four dimensions: 1) ‘Individual knowledge and skills’ (items 1–6), 2) ‘Individual attitudes and beliefs’ (items 7–10), 3) ‘Organizational support’ (items11–13), and 4) ‘Organizational resources’ (items 14–17). Items 1–10 are rated from 0 = ‘none’ to 10 = ‘high’, while items 11–17 ranged from 0 = ‘totally disagree’ to 10 = ‘totally agree’. Higher scores across subscales indicate greater support for and readiness to implement smoking cessation practices^17^. For ease of interpretation, in this study all subscale and total scores were rescaled to a common 0–10 range (0 = lowest; 10 = highest); items 14–17 were reverse scored so that higher values consistently indicate greater organizational resources.

The Spanish version of the KABO_S was validated in a sample of 511 health professions students, demonstrating good psychometric properties with an overall Cronbach’s alpha coefficient of 0.83, and subscale scores ranging from 0.81 to 0.92. The English version, tested in 186 students, also showed strong internal consistency, with an overall Cronbach’s alpha of 0.88, and subscale values between 0.82 to 0.94. In both versions, corrected item-total correlations were acceptable for all subscales (r>0.30)^17^.

### Procedure

After providing informed consent, participating health professions students completed a self-administered baseline questionnaire, including sociodemographic data and the KABO_S instrument, between February and April 2021. Students then accessed the INSTrUCT Online Brief Intervention for Smoking Cessation Training Program and repeated the KABO_S questionnaire again one month after completion. Both pre- and post-intervention KABO_S questionnaires were available online through the program’s platform. Up to five follow-up reminders were sent via email to encourage completion of the post-questionnaire. Throughout the study, participants had access to information, support, and guidance from a designated member of the research team at each participating institution.

### Outcome measures

The main outcome measures were each of the four KABO_S dimensions: 1) Individual knowledge and skills (IKS), 2) Individual attitudes and beliefs (IAB), 3) Organizational support (OS), and 4) Organizational resources (OR), with scores ranging from 0 to 10, measured at baseline and one-month post-intervention. The overall KABO_S score was calculated as the sum of the four-dimension scores. Scores ≥5 were considered indicative of an adequate level of support for and implementation of smoking cessation practices; whereas scores <5 were considered inadequate.

Covariates were sociodemographic characteristics, including gender (female, male, or other), age, and country (Spain or the UK); tobacco use (user or non-user); and whether the participants had received previous training in smoking cessation (yes or no). Tobacco use was self-reported and classified based on current use of tobacco products at the time of the survey.

### Potential covariates

Potential covariates were assessed at baseline and selected *a priori* based on their plausible association with baseline KABO_S scores and responsiveness to the educational intervention. The baseline questionnaire collected sociodemographic variables (sex and age) and tobacco use status. These variables (sex, age, and tobacco use) were included as covariates in the adjusted models.

### Statistical analysis

Descriptive analyses were calculated for the overall KABO_S and each of its four dimensions. Categorical variables are presented as frequencies and percentages, and continuous variables as mean and standard deviation (SD), or median and interquartile range (IQR), as appropriate. Ninety-five percent confidence intervals (95% CIs) were calculated using distribution-based methods, applying exact binomial confidence intervals for proportions and t-based confidence intervals for means, in order to quantify the precision of the estimates. Pre–post differences were initially assessed using Student’s paired t-test (two-tailed). Paired Cohen’s d was calculated to estimate the effect size of the training. Generalized linear mixed models (GLMMs) were used to assess pre-post changes in the overall KABO_S scores and their subscales, accounting for participant-level clustering and adjusting for gender, age, and tobacco use. In the models, all KABO_S scores were rescaled to a common 0-10 range. Statistical significance was set at p<0.05. Analyses were conducted with RStudio (version 4.3.0).

### Ethical considerations

The research protocol was approved by the Research Ethics Committee of the Hospital Universitari de Bellvitge (reference number PR389/19, Date: 19 December 2019) and accepted by all participating institutions. All participants voluntarily provided written informed consent prior to participation.

## RESULTS

### Description of the sample

A total of 756 health professions students participated in the study, 652 from Spain and 104 from the UK. The majority were females (87.8%) and under 22 years old (80.9%). Tobacco use was reported by 26.3% of participants, and 11.0% had previously received training in smoking cessation interventions.

### Changes in knowledge, attitudes, behaviors, and organizational factors before and one month after the training program

Statistically significant increases were observed in the overall KABO_S score and all four dimensions in both countries from baseline to one month after completing the training program (Table 2). The mean overall KABO_S score increased from 4.82 (SD=1.34) at baseline to 6.69 (SD= 1.26) at one month (p<0.01). By dimension, mean scores increased from 3.38 (SD=2.02) to 6.88 (SD=1.62) on individual knowledge and skills (p<0.01), from 6.31 (SD=2.01) to 7.78 (SD=1.55) on individual attitudes and beliefs (p<0.01), from 4.24 (SD=2.63) to 5.46 (SD=2.96) on organizational support (p<0.01), and from 5.94 (SD=2.31) to 6.25 (SD=2.45) on organizational resources (p<0.01).

**Table 1:** Sociodemographic characteristics of undergraduate health professions students in Spain (N=652) and the UK (N=104), January 2020–June 2022 (N=756)

*Characteristics*	*n (%)*	*95% CI^†^*
Gender*		
Male	90 (11.9)	9.60–14.2
Female	664 (85.5)	85.5–90.1
**Age** (years), mean (SD)	21.6 (4.79)	21.3–21.9
**Age** (years)		
<21.6	611 (80.9)	78.1–83.7
≥21.6	144 (19.1)	16.3–21.9
**Tobacco use**		
Yes	199 (26.3)	23.2–29.4
**Previous training**		
Yes	83 (11.0)	8.80–13.2
**Country**		
Spain	652 (86.2)	83.7–88.7
United Kingdom	104 (13.8)	11.3–16.3

* There were also 2 participants with other gender.

† 95% CIs were calculated in R using binomial methods.

**Table 2:** Knowledge, attitudes, behaviors, and organizational factors related to competent smoking cessation practice among undergraduate health professions students in Spain (N=652) and the United Kingdom (N=104), a pre–post study assessing outcomes before and 1 month after completion of an online brief smoking intervention training, January 2020–June 2022 (N=756)

	*Baseline*		*Month 1*		p *
	*Mean (SD)*	*95% CI*	*Mean (SD)*	*95% CI*	
Overall KABO_S	4.82 (1.34)	4.72–4.91	6.69 (1.26)	6.60–6.78	<0.01
Individual knowledge and skills (IKS)	3.38 (2.02)	3.23–3.52	6.88 (1.62)	6.76–6.99	<0.01
Individual attitudes and beliefs (IAB)	6.31 (2.01)	6.17–6.45	7.78 (1.55)	7.67–7.89	<0.01
Organizational support (OS)	4.24 (2.63)	4.05–4.43	5.46 (2.96)	5.25–5.67	<0.01
Organizational resources (OR)	5.94 (2.31)	5.77–6.01	6.25 (2.45)	6.07–6.43	<0.01

* Student’s t-test. Subscale and total scores were rescaled to a 0–10 range for comparability (higher scores indicate higher levels of the construct). Raw total score range: 17–170.

In the Spanish sample, the mean overall KABO_S score increased from 4.92 (SD=1.32) to 6.70 (SD=1.25), with a median improvement of 35.8% (interquartile range, IQR: 18.3–51.4) and an effect size of 1.39 (95% CI: 1.26–1.51) (Table 3). By dimension, individual knowledge and skills, and individual attitudes and beliefs showed the greatest increases. Individual knowledge and skills rose from 3.34 (SD=2.03) to 6.74 (SD=1.58), with a median improvement of 52.7% (IQR: 33.7–67.8) and an effect size of 1.87 (95% CI: 1.70–2.03). Individual attitudes and beliefs increased from 6.47 (SD=1.95) to 7.75 (SD=1.56), with a median improvement of 38.5% (IQR: 0.0–63.2) and an effect size of 0.72 (95% CI: 0.63–0.82). Organizational resources showed the smallest improvement, increasing from 6.08 (SD=2.23) to 6.41 (SD=2.32), with a median improvement of 15.2% (IQR: -28.8–50.0) and an effect size of 0.15 (95% CI: 0.06–0.24).

**Table 3:** Pre–post changes in overall KABO_S questionnaire scores and its four dimensions, by country, among undergraduate health professions students in Spain (N=652) and the United Kingdom (N=104), a pre– post study assessing outcomes before and 1 month after completion of an online brief smoking intervention training, January 2020–June 2022 (N=756)

	*n*	*Baseline*	*Month 1*	*Percent improvement* *achieved*		*Effect size* *(95% CI)*	p*
		*Mean (SD)*	*Mean (SD)*	*Median*	*IQR*		
**Spain**							
Overall KABO_S	652	4.92 (1.32)	6.70 (1.25)	35.8	18.3–51.4	1.39 (1.26–1.51)	<0.01
Individual knowledge and skills (IKS)	651	3.34 (2.03)	6.74 (1.58)	52.7	33.7–67.8	1.87 (1.70–2.03)	<0.01
Individual attitudes and beliefs (IAB)	643	6.47 (1.95)	7.75 (1.56)	38.5	0.0–63.2	0.72 (0.63–0.82)	<0.01
Organizational support (OS)	636	4.46 (2.61)	5.59 (2.92)	22.2	-20.0–60.0	0.41 (0.31–0.50)	<0.01
Organizational resources (OR)	592	6.08 (2.23)	6.41 (2.32)	15.2	-28.8–50.0	0.15 (0.06–0.24)	<0.01
**United Kingdom**							
Overall KABO_S	104	4.23 (1.36)	6.67 (1.27)	42.0	29.7–56.4	1.84 (1.46–2.23)	<0.01
Individual knowledge and skills (IKS)	103	3.62 (1.97)	7.76 (1.60)	69.1	50.0–85.0	2.3 (1.82–2.77)	<0.01
Individual attitudes and beliefs (IAB)	104	5.33 (2.11)	7.94 (1.49)	60.8	21.1–78.9	1.43 (1.05–1.80)	<0.01
Organizational support (OS)	104	2.87 (2.30)	4.70 (3.09)	22.1	0.0–53.5	0.66 (0.44–0.88)	<0.01
Organizational resources (OR)	98	5.07 (2.64)	5.24 (3.01)	5.7	-31.6–50.0	0.06 (-0.80–0.30)	0.628

* Student’s t-test. Subscale and total scores were rescaled to a 0–10 range for comparability (higher scores indicate higher levels of the construct). Raw total score range: 17–170. IQR: interquartile range.

In the sample from the UK, the mean overall KABO_S score increased from 4.23 (SD=1.36) to 6.67 (SD=1.27), with a median improvement of 42.0% (IQR: 29.7–56.4) and an effect size of 1.84 (95% CI: 1.46 – 2.23) (Table 3). As in Spain, the greatest improvements were observed in individual knowledge and skills, and individual attitudes and beliefs. Individual knowledge and skills increased from 3.62 (SD=1.97) to 7.76 (SD=1.60), with a median improvement of 69.1% (IQR: 50.0–85.0) and an effect size of 2.3 (95% CI: 1.82–2.77), and individual attitudes and beliefs increased from 5.33 (SD=2.11) to 7.94 (SD=1.49) with a median improvement of 60.8% (IQR: 21.1–78.9) and an effect size of 1.43 (95% CI: 1.05–1.80). Organizational resources again showed the smallest improvement, increasing from 5.07 (SD=2.64) to 5.24 (SD=3.01), with a median improvement of 5.7% (IQR: -31.6–50.0) and an effect size of 0.06 (95% CI: -0.80–0.30).

### Effect of the training program on knowledge, attitudes, behaviors, and organizational factors over time

The significant effects of time observed in the previous analysis for the overall KABO_S score and its four dimensions were confirmed by the generalized linear mixed models (Tables 4 and 5). One month after the training, the overall KABO_S score increased significantly in participants from both Spain (β=1.78; 95% CI: 1.67–1.90) (Table 4) and the UK (β=2.44; 95% CI: 2.13–2.74) (Table 5). Similar positive effects were observed across all four dimensions. For individual knowledge and skills, the increase was β=3.40 (95% CI: 3.22–3.58) for participants from Spain and β=4.14 (95% CI: 3.69–4.59) for participants from the UK. For individual attitudes and beliefs, β=1.29 (95% CI: 1.13–1.44) in participants from Spain and β=2.61 (95% CI: 2.13–3.09) in participants from the UK. For organizational support, the increase was β=1.13 (95% CI: 0.88–1.38) for participants from Spain and β=1.83 (95% CI: 1.27–2.39) for participants from the UK. For organizational resources, participants from both countries showed modest increases (Spain: β=0.33; 95% CI: 0.13–0.53; the UK: β=0.17; 95% CI: -0.51–0.85). Among participants from both countries, no significant changes/interactions were found between time and gender, age, or tobacco use.

**Table 4:** Effect of an online brief smoking intervention training on overall KABO_S questionnaire scores and its four dimensions according to independent variables among undergraduate health professions students in Spain, a pre–post study with assessment before and 1 month after training January 2020–June 2022 (N=652)

	*Overall KABO_S*		*Individual* *knowledge and* *skills*		*Individual* *attitudes and* *beliefs*		*Organizational* *support*		*Organizational* *resources*	
	*β*	*95% CI*	*β*	*95% CI*	*β*	*95% CI*	*β*	*95% CI*	*β*	*95% CI*
Crude Model*										
Intercept	4.92	4.82–5.01	3.34	3.20–3.48	6.47	6.33–6.60	4.46	4.24–4.67	6.08	5.90–6.25
Month 1 vs Baseline	1.78	1.67–1.90	3.40	3.22–3.58	1.29	1.13–1.44	1.13	0.88–1.38	0.33	0.13–0.53
Adjusted Model*										
Intercept	4.72	4.24–5.21	3.26	2.61–3.90	5.89	5.22–6.56	4.03	2.99–5.08	6.27	2.99–5.08
Month 1 vs Baseline (ref.)	1.78	1.67–1.90	3.40	3.22–3.59	1.29	1.13–1.45	1.13	0.88–1.38	0.34	0.13–0.54
Females vs Males (ref.)	0.06	-0.18–0.30	-0.01	-0.33–0.30	0.29	-0.04–0.63	-0.25	-0.77–0.27	0.18	-0.25–0.61
Age (years)	0.01	-0.01–0.03	0.01	-0.02–0.03	0.02	-0.01–0.04	0.03	-0.01–0.07	-0.01	-
Tobacco use vs non-tobacco use (ref.)	-0.09	-0.27–0.09	-0.15	-0.39–0.09	-0.03	-0.28–0.22	0.03	-0.36–0.42	-0.14	-

* Generalized linear mixed model 0 to 10 (units of increase). β: fixed-effect regression coefficient from the GLMM on the link scale. Adjusted for time, sex, age, and tobacco use.

**Table 5:** Effect of an online brief smoking intervention training on overall KABO_S questionnaire scores and its four dimensions according to independent variables among undergraduate health professions students in the United Kingdom, a pre–post study with assessment before and 1 month after training, January 2020–June 2022 (N=104)

	*Overall KABO_S*		*Individual* *knowledge and* *skills*		*Individual* *attitudes and* *beliefs*		*Organizational* *support*		*Organizational* *resources*	
	*β*	*95% CI*	*β*	*95% CI*	*β*	*95% CI*	*β*	*95% CI*	*β*	*95% CI*
Crude Model*										
Intercept	4.23	3.98–4.49	3.63	3.28–3.97	5.33	4.98–5.69	2.87	2.34–3.40	5.07	4.52–5.61
Month 1 vs Baseline	2.44	2.13–2.74	4.14	3.69–4.59	2.61	2.13–3.09	1.83	1.27–2.39	0.17	-0.51–0.85
Adjusted Model*										
Intercept	4.88	3.71–6.06	4.75	3.24–6.27	5.73	4.23–7.22	3.57	1.00–6.14	5.22	2.78–7.66
Month 1 vs Baseline (ref.)	2.42	2.11–2.72	4.11	3.66–4.56	2.61	2.12–3.09	1.81	1.25–2.38	0.13	-0.55–0.82
Females vs Males (ref.)	-0.32	-1.29–0.64	-0.81	-2.05–0.43	-0.13	-1.36–1.09	-1.38	-3.49–0.73	1.02	-0.98–3.02
Age (years)	-0.01	-0.04–0.02	-0.02	-0.06–0.02	-0.01	-0.05–0.03	0.02	-0.04–0.09	-0.04	-0.10–0.02
Tobacco use vs non-tobacco use (ref.)	0.05	-0.47–0.56	0.37	-0.29–1.04	0.20	-0.46–0.85	0.14	-0.98–1.27	-0.66	-1.73–0.41

* Generalized linear mixed model 0 to 10 (units of increase). β: fixed-effect regression coefficient from the GLMM on the link scale. Adjusted for time, sex, age, and tobacco use.

## DISCUSSION

To our knowledge, this is the first large-scale study to evaluate a smoking cessation training program for health professions students using a validated, multidimensional instrument that incorporates organizational readiness as a core outcome. Our findings showed that self-reported knowledge, attitudes, behaviors, and organizational factors significantly improved among health professions students in Spain and the UK one month after completing the INSTrUCT Online Brief Intervention for Smoking Cessation Training Program. There was a marked increase in the overall KABO_S scores, as well as in each of its four dimensions, over time. The observed improvements remained after adjustment for gender, age, and tobacco use, suggesting that the findings were robust to these covariates. These improvements not only support but also extend previous evidence regarding the effectiveness of the training developed within the INSTrUCT project^18^. In addition to enhancing core competences – knowledge, attitudes, and skills – the training program contributed to more positive self-reported behaviors and improved perceptions of organizational factors that support smoking cessation during students’ education. In doing so, it provides new evidence on the effectiveness and scalability of online cessation training in healthcare professions education.

E-learning in smoking cessation training for health professionals has proven highly effective in increasing tobacco-related knowledge^21,22^, often achieving outcomes comparable to face-to-face methods such as role-playing^19,23,24^. However, the online training program evaluated in this study stands out for its comprehensive focus on multiple components that influence the delivery of smoking cessation interventions, including not only knowledge and attitudes but also skills and behaviors. It is also one of the few programs shown to improve self-efficacy and competence in smoking cessation practices^18^.

To evaluate these broad dimensions, the use of the KABO_S questionnaire was particularly relevant. Unlike other tools that assess tobacco-related knowledge, attitudes, and behaviors by focusing mainly on cognitive and behavioral components^25,26^, the KABO_S questionnaire evaluates self-perceived competence and readiness to act. It measures levels of preparation, motivation, and familiarity with pharmacological treatments, while also capturing students’ perceptions of organizational support and available resources – factors known to be critical for implementation^27,28^. Supporting this, the review of Li et al.^28^ found that nurses’ self-perceived competences and perceived organizational factors are positively associated with the actual delivery of smoking cessation care. However, this is the first study to explore these factors among health professions students. This is particularly relevant, as clinical placements provide a valuable opportunity to apply classroom learning in real-world settings^29^, helping to bridge the gap between knowledge and practice^30^.

Not surprisingly, organizational factors, specifically the resources available to support implementation of smoking cessation interventions, showed the least improvement, as this study did not involve institutional-level organizational changes. However, in order to support integration of the training into organizations and/or academic settings, various supporting materials were developed, including workshops, training activities, a teacher’s manual, and additional resources^31^. In line with this, other studies have noted that organizational factors often act as moderators of training effectiveness, rather than being directly influenced by training alone^32^. While such factors may be less amenable to change through student or staff training alone, improvements can occur when interventions target the health system level and include structural organizational modifications such as updated protocols, documentation systems, and positive incentives^33^. These findings suggest that academic and healthcare institutions hosting student placements should adopt strategies to strengthen organizational resources; thereby improving the effectiveness and sustainability of training initiatives, and enhancing student engagement and learning^32,33^.

Importantly, despite including students with varying sociodemographic characteristics (gender, age, and country) and professional backgrounds (nursing, medicine, pharmacy, and psychology), results were largely consistent across subgroups. This consistency supports the quality and potential universality of the training program. Nevertheless, further research is needed to explore the small differences observed between the Spanish and the UK samples. Specifically, the training program had a greater effect on the overall KABO_S score, and most of its dimensions were greater in the UK than in Spain, except for organizational resources. Moreover, the current results reflect only short-term effects (one month after the training).

The findings of this study, therefore, highlight the potential impact of such a training program to enhance health professions students’ ability to implement smoking cessation interventions in future practice. Supporting this, an online training conducted in Catalonia (a region of Spain), which introduced organizational changes to support smoking cessation, found a significant positive association between increased individual skills and organizational support with higher implementation rates of the 5As Brief Tobacco Intervention Model^33^. Similarly, other educational programs targeting health and nursing professionals have shown improved implementation and documentation of smoking cessation interventions^34,35^. For instance, Gennette et al.^34^ implemented a face-to-face educational program aimed at improving smoking cessation documentation among 11 health professionals in the USA. Six months post-intervention, documentation compliance increased from 25% to 77%. In another study, Mizuno et al.^35^ implemented a web-based educational program on smoking cessation counselling for 678 Japanese nurses. Three months after the intervention, they found increased odds of implementing the 5As Brief Tobacco Intervention Model had increased, specifically the Advice, Assess, and Assist components, compared with baseline.

Nonetheless, further research is needed to assess the long-term effects of such training programs on students’ future clinical practice. Beyond self-reported data, the use of more objective measures, such as students’ practice patterns and patient smoking cessation outcomes^11^, would be valuable for estimating the true impact of improvements in self-reported knowledge, attitudes, behaviors, and organizational factors. For example, a systematic review and meta-analysis of 28 randomized controlled trials evaluating tobacco dependence education for health professional students found that, at six months, smoking cessation rates were higher among smokers counselled by trained students than among those in the control group^11^.

### Strength and limitations

This study has several strengths. It evaluates a brief, scalable smoking cessation training program embedded in health professions education and uses a within-participant pre–post design that is well suited to detecting change over time in the same individuals. Although the absence of a control group limits causal inference, the paired approach reduces between-subject heterogeneity and allows participants to serve as their own baseline. The use of the psychometrically validated KABO_S instrument (available in both Spanish and English) supports the credibility and consistency of the measurements across educational contexts. In addition, generalized linear mixed models account for within-participant correlation and adjust for key measured covariates (sex, age, and tobacco use), strengthening the robustness of the estimates. Finally, reporting results separately by country enhances transparency and facilitates interpretation of potential contextual differences.

This study presents several limitations that should be considered when interpreting the results. First, as is common in educational intervention studies, the lack of a control group restricts the ability to establish a direct causal relationship between the training program and the observed changes. Nevertheless, the pre-post design using paired analyses offers a reasonable degree of internal validity by accounting for individual baseline differences. In addition, the linear mixed models were adjusted for all measured sociodemographic and tobacco use variables: gender, age, and tobacco use, which adds robustness to the interpretation of the findings.

Second, due to the voluntary nature of participation, students who chose to take part may have had a greater interest in tobacco prevention and therefore been more motivated to score highly on the questionnaires. This potential selection bias may have influenced the overestimation of the impact of the program.

Third, residual confounding cannot be fully excluded. For instance, although the year of study was available, it showed limited variability in our sample; approximately 97% of participants were in intermediate years, and the mean age was 21 years, suggesting a broadly comparable academic stage across the cohort. Given that the academic year is closely related to age (e.g. most students in Spain enter university at approximately 18 years), age adjustment is expected to account, at least partially, for differences in academic progression. Furthermore, although UK students were slightly older on average, they were enrolled in the same course year/module in which the mandatory training was delivered. Therefore, the year of study is unlikely to explain the observed differences between UK and Spanish students. In contrast, socioeconomic status and degree program characteristics were not available in an analyzable form and therefore could not be included as covariates, and may partially contribute to unmeasured differences between cohorts. Additionally, although models were adjusted for tobacco use status, we did not account for other smoking-related factors that may influence baseline scores and responsiveness to the intervention, such as smoking intensity (e.g. cigarettes per day) and years of smoking, which could also act as residual confounders. In addition, generalizability may be limited because students from Belgium and Portugal were excluded from the present analysis due to the lack of validated French and Portuguese versions of the KABO_S instrument. Moreover, the sample was imbalanced between countries, which may affect the precision and comparability of country-specific estimates; to increase transparency, results are presented separately by country in several tables.

Fourth, outcomes were analyzed at the domain level of the KABO_S instrument, and we did not conduct item-level analyses to determine which specific components within each domain improved over time. In addition, as the videos were intended primarily to support observational learning, we did not collect objective measures of counselling performance linked to the educational videos (e.g. graded recordings, rubric-based ratings, OSCE scores, or platform performance metrics). Therefore, we were unable to examine whether improvements in specific learning components were associated with ‘video counselling’ skills. Moreover, while completion of all training components was objectively verified, individual time-on-task data were not available. Consequently, we were unable to explore potential dose–response relationships between the amount of time spent on the program and changes in knowledge, attitudes, behaviors, or organizational perceptions. Furthermore, we did not include objective measures of clinical application (e.g. observed counselling performance in real clinical settings), which limits the ability to conclude the translation of these improvements into clinical practice.

Finally, all data were self-reported, which may be affected by social desirability or recall bias. However, the use of a psychometrically validated instrument (KABO_S), available in both Spanish and English, enhances the credibility and consistency of the measurements and supports the reliability of the results across diverse educational contexts.

## CONCLUSIONS

This study found a significant improvement in self-reported knowledge, attitudes, behaviors, and organizational factors among health professions students from Spain and the UK after completing the INSTrUCT Online Brief Intervention for Smoking Cessation Training Program. These findings suggest that incorporating such training into health professions education may offer potential benefits and warrant further evaluation. However, further research is needed to assess its long-term effects on students’ self-reported outcomes and to determine the extent to which these improvements influence clinical practice. The dissemination of this training across Europe and internationally is paramount to help reduce the future burden of tobacco-related diseases and to equip the next generation of health professionals to become leaders in tobacco prevention and cessation.

## Data Availability

The data supporting this research are available from the authors on reasonable request.

## References

[1] Dai X, Gakidou E, Lopez AD. Evolution of the global smoking epidemic over the past half century: Strengthening the evidence base for policy action. Tob Control. 2022;31(2):129-137. doi:10.1136/tobaccocontrol-2021-056535 35241576

[2] World Health Organization. WHO global report on trends in prevalence of tobacco use 2000–2030. Accessed January 14, 2026. https://www.who.int/publications/i/item/9789240088283

[3] Nilan K, McKeever TM, McNeill A, Raw M, Murray RL. Prevalence of tobacco use in healthcare workers: A systematic review and meta-analysis. PLoS One. 2019;14(7):e0220168. doi:10.1371/journal.pone.0220168 31344083 PMC6657871

[4] World Health Organization. Tobacco. Accessed January 14, 2026. https://www.who.int/europe/health-topics/tobacco#tab=tab_2

[5] World Health Organization. The role of healthcare professionals in tobacco control. Accessed January 14, 2026. https://apps.who.int/iris/handle/10665/43219

[6] World Health Organization. WHO report on the global tobacco epidemic, 2023: protect people from tobacco smoke; 2023. Accessed January 14, 2026. https://www.who.int/publications/i/item/9789240077164

[7] Duaso M, Duncan D. Health impact of smoking and smoking cessation strategies: Current evidence. Br J Community Nurs. 2012;17(8):356-363. doi:10.12968/bjcn.2012.17.8.356 22875209

[8] Babb S, Malarcher A, Schauer G, Asman K, Jamal A. Quitting smoking among adults—United States, 2000-2015. MMWR Morb Mortal Wkly Rep. 2017;65(52):1457-1464. doi:10.15585/mmwr.mm6552a1 28056007

[9] Rigotti NA, Kruse GR, Livingstone-Banks J, Hartmann-Boyce J. Treatment of tobacco smoking: A review. JAMA. 2022;327(6):566-577. doi:10.1001/jama.2022.0395 35133411

[10] Fukada M. Nursing competency: Definition, structure and development. Yonago Acta Med. 2018;61(1):1-7. doi:10.33160/yam.2018.03.001 29599616 PMC5871720

[11] Hyndman K, Thomas RE, Schira HR, et al. The effectiveness of tobacco dependence education in health professional students’ practice: A systematic review and meta-analysis of randomized controlled trials. Int J Environ Res Public Health. 2019;16(21):4158. doi:10.3390/ijerph16214158 31661922 PMC6862178

[12] Melzer AC, Reese ZA, Mascarhenas L, et al. Education for tobacco use disorder treatment: Current state, evidence, and unmet needs. ATS Sch. 2023;4(4):546-566. doi:10.34197/ats-scholar.2022-0131RE 38196686 PMC10773493

[13] Schultz ASH, Dunford D, Atout R, Grymonpre R. Situating tobacco dependency education in health professional prelicensure curricula: An interprofessional learning opportunity. Can J Respir Ther. 2015;51(4):86-88. Accessed January 14, 2026. https://pubmed.ncbi.nlm.nih.gov/26566378/26566378 PMC4631134

[14] Čivljak M, Ačkar L, Puljak L. The knowledge, attitudes and behaviors of hospital nurses on smoking cessation interventions: A cross-sectional study. BMC Nurs. 2023;22(1):228. doi:10.1186/s12912-023-01394-7 37394472 PMC10316570

[15] Sreeramareddy CT, Ramakrishnareddy N, Rahman M, Mir IA. Prevalence of tobacco use and perceptions of student health professionals about cessation training: Results from Global Health Professions Students Survey. BMJ Open. 2018;8(5):e017477. doi:10.1136/bmjopen-2017-017477 PMC5988057 29804056

[16] Westmaas JL, Kates I, Makaroff L, Henson R. Barriers to helping patients quit smoking. Public Health Pract (Oxf). 2023;6:100409. doi:10.1016/j.puhip.2023.100409 37554288 PMC10405087

[17] Pueyo-Garrigues M, Agüera Z, Andrés A, et al. Knowledge, attitudes, behavioral and organizational factors of health professions students for a competent smoking cessation practice. Nurse Educ Pract. 2023;70:103647. doi:10.1016/j.nepr.2023.103647 37121026

[18] Pardavila-Belio MI, Moreno-Arroyo C, Romero-Clará O, et al. Adaptation, implementation, and evaluation of an online health sciences training program for brief smoking intervention. Nurse Educ Today. 2023;130:105924. doi:10.1016/j.nedt.2023.105924 37677986

[19] Milella MS, Sansone A, Basili S, et al. E-learning course improves knowledge in tobacco dependence, electronic nicotine delivery systems and heat-not-burn products in medical school students. Clin Ter. 2021;172(5):427-434. doi:10.7417/CT.2021.2353 34625774

[20] Andrés A, Castellano Y, Fu M, et al. Exploring individual and contextual factors contributing to tobacco cessation intervention implementation. Addict Behav. 2018;88:163-168. doi:10.1016/j.addbeh.2018.08.003 30205255

[21] Martínez C, Castellano Y, Company A, et al. Impact of an online training program in hospital workers’ smoking cessation interventions in Bolivia, Guatemala and Paraguay. Gac Sanit. 2018;32(3):236-243. doi:10.1016/j.gaceta.2017.10.020 29398107

[22] Martínez C, Castellano Y, Andrés A, et al. Impact of an online training program in smoking cessation interventions in hospitals. J Nurs Scholarsh. 2019;51(4):449-458. doi:10.1111/jnu.12469 30874373

[23] Lauerer E, Tiedemann E, Polak T, Simmenroth A. Can smoking cessation be taught online? Int J Med Educ. 2021;12:12-21. doi:10.5116/ijme.5ff9.bccc 33507877 PMC7883797

[24] Stolz D, Langewitz W, Meyer A, et al. Enhanced didactic methods of smoking cessation training for medical students: A randomized study. Nicotine Tob Res. 2012;14(2):224-228. doi:10.1093/ntr/ntr186 22090454

[25] Campo L, Vecera F, Fustinoni S. Validation of a questionnaire to assess smoking habits among university students. Int J Environ Res Public Health. 2021;18(22):11873. doi:10.3390/ijerph182211873 34831630 PMC8621372

[26] Martínez C, Castellano Y, Laroussy K, et al. Knowledge, attitudes, and training in tobacco dependence and cessation treatment among nursing students in Catalonia. Int J Ment Health Addict. 2023;21(2):1041-1056. doi:10.1007/s11469-021-00640-w 37261115 PMC10229109

[27] Martínez C, Castellano Y, Andrés A, et al. Factors associated with implementation of the 5A’s smoking cessation model. Tob Induc Dis. 2017;15:41. doi:10.1186/s12971-017-0146-7 29142531 PMC5669025

[28] Li M, Koide K, Tanaka M, et al. Factors associated with nursing interventions for smoking cessation: A narrative review. Nurs Rep. 2021;11(1):64-74. doi:10.3390/nursrep11010007 34968313 PMC8608102

[29] Ravik M, Bjerkelund GM, Hvalvik S, Reierson IÅ. Student nurses’ learning of practical skills in hospital placements. Nurse Educ Pract. 2025;83:104275. doi:10.1016/j.nepr.2025.104275 39892252

[30] Martínez C, Camarelles Guillem F, González-Viana A, Sánchez Á, Tigova O, Fernández E. De la evidencia a la práctica: la ciencia de la diseminación e implementación en atención primaria y comunitaria. Aten Primaria. 2025;57(1):103077. doi:10.1016/j.aprim.2024.103077 39265319 PMC11415847

[31] Pardavila-Belio MI, Conga-Armayor N, Pueyo-Garrigues M, et al. INSTrUCT: An open educational resource in brief intervention on smoking in university students. Pamplona: Ediciones Universidad de Navarra; 2021. Accessed January 14, 2026. https://ebooks.eunsa.es/library/publication/instruct-an-open-educational-resource-in-brief-intervention-on-smoking-in-university-students

[32] Le K, Chen TA, Martinez Leal I, et al. Organizational-level moderators impacting tobacco-related knowledge change after tobacco education training in substance use treatment centers. Int J Environ Res Public Health. 2021;18(14):7597. doi:10.3390/ijerph18147597 34300052 PMC8305177

[33] Martínez C, Feliu A, Enriquez M, et al. Improving tobacco cessation interventions in hospitals: Pre-post evaluation of an innovative health systems intervention in Catalonia (Spain). Transl Behav Med. 2024;14(9):549-560. doi:10.1093/tbm/ibae016 38916135

[34] Gennette RL. Improving smoking cessation education and documentation. J Am Assoc Nurse Pract. 2021;34(2):357-363. doi:10.1097/JXX.0000000000000630 34469361

[35] Mizuno M, Yagasaki K, Imai Y, et al. Impact of a web-based educational program on Japanese nurses’ tobacco cessation practice and attitudes in oncology settings. J Nurs Scholarsh. 2022;54(3):315-323. doi:10.1111/jnu.12733 34750960

